# The Compound 2-Hexyl, 5-Propyl Resorcinol Has a Key Role in Biofilm Formation by the Biocontrol Rhizobacterium *Pseudomonas chlororaphis* PCL1606

**DOI:** 10.3389/fmicb.2019.00396

**Published:** 2019-02-28

**Authors:** Claudia E. Calderón, Sandra Tienda, Zaira Heredia-Ponce, Eva Arrebola, Gerardo Cárcamo-Oyarce, Leo Eberl, Francisco M. Cazorla

**Affiliations:** ^1^Departamento de Microbiología, Facultad de Ciencias, Universidad de Málaga, Málaga, Spain; ^2^Instituto de Hortofruticultura Subtropical y Mediterránea “La Mayora,” Consejo Superior de Investigaciones Científicas, Universidad de Málaga, IHSM-UMA-CSIC, Málaga, Spain; ^3^Department of Plant and Microbial Biology, University of Zürich, Zurich, Switzerland

**Keywords:** antifungal, biocontrol, biofilm, motility, adhesion, confocal laser scanning microscopy

## Abstract

The production of the compound 2-hexyl-5-propyl resorcinol (HPR) by the biocontrol rhizobacterium *Pseudomonas chlororaphis* PCL1606 (PcPCL1606) is crucial for fungal antagonism and biocontrol activity that protects plants against the phytopathogenic fungus *Rosellinia necatrix*. The production of HPR is also involved in avocado root colonization during the biocontrol process. This pleiotrophic response prompted us to study the potential role of HPR production in biofilm formation. The swimming motility of PcPLL1606 is enhanced by the disruption of HPR production. Mutants impaired in HPR production, revealed that adhesion, colony morphology, and typical air–liquid interphase pellicles were all dependent on HPR production. The role of HPR production in biofilm architecture was also analyzed in flow chamber experiments. These experiments revealed that the HPR mutant cells had less tight unions than those producing HPR, suggesting an involvement of HPR in the production of the biofilm matrix.

## Introduction

Members of the genus *Pseudomonas* possess a substantial amount of metabolic diversity, and many of them are able to colonize a wide range of niches (Madigan and Martinko, 2015). *Pseudomonas* spp. can produce a variety of metabolites, many of them inhibitory to other microorganism and are also involved in the biological control of plant pathogens (Morrissey et al., 2004; Gross and Loper, 2009; Raaijmakers and Mazzola, 2012). The biocontrol mechanisms that *Pseudomonas* spp. display against soilborne plant pathogens include the production of antibiotics, hydrolytic enzymes, and volatile organic compounds, competition for space and nutrients, and the induction of systematic resistance in plants (Raza et al., 2013; Faheem et al., 2015; Yunus et al., 2016). Highly in depth research has been performed on the exploration and identification of the antibiotics produced by different *Pseudomonas* spp., biocontrol pseudomonads frequently produce more than one antimicrobial compound (Haas and Keel, 2003). The most well-known antibiotics produced by *Pseudomonas* spp. include 2,4-diacetylphloroglucinol (2,4-DAPG), phenazines (PHZs), pyrrolnitrin (PRN), pyoluteorin (PLT), hydrogen cyanide (HCN), and 2-hexyl-5-propyl resorcinol (HPR; Cazorla et al., 2006; Gross and Loper, 2009). These antibiotics could be directly involved in other different phenotypes related to biocontrol ability in addition to antagonism, such as plant growth promotion and niche competition (Weller, 2007; Ramette et al., 2011; Wang et al., 2015; Raio et al., 2017).

*Pseudomonas chlororaphis* PCL1606 (PcPCL1606) is a biocontrol agent able to suppress plant diseases caused by different soilborne phytopathogenic fungi (Cazorla et al., 2006). Previous studies revealed that this rhizobacterium produces the three antifungal compounds HPR, PRN, and HCN; however, only HPR has been demonstrated to be directly involved in the antagonism and the biocontrol ability of this strain (Calderón et al., 2015). HPR is a small molecule, which belongs to the group of alkyresorcinols, produced by different bacteria. This compound is liberated from the cell to the environment, where display some antimicrobial activity (Nowak-Thompson et al., 2003). The genes responsible for HPR production, the *dar* genes, which were previously identified in *P. chlororaphis* subsp. *aurantiaca* BL915 (Nowak-Thompson et al., 2003), have also been demonstrated to be present in strain PcPCL1606 (Calderón et al., 2013, 2014a). In PcPCL1606, the *dar* genes are located in a cluster containing three biosynthetic genes (*darA*, *darB*, and *darC*), followed by two transcriptional regulatory genes *darS* and *darR*, both belonging to the transcriptional regulatory family AraC/XylS. In addition, it has been demonstrated that the biosynthetic genes are positively regulated by the DarSR transcriptional regulators, and all the *dar* genes are under the control of GacS (Calderón et al., 2014a). In addition, some alkylresorcinols (to which the compound HPR belongs) can be proposed to be possible signal molecules in the genus *Photorhabdus* (Brameyer et al., 2015).

It was demonstrated that HPR production is involved in the multitrophic avocado root-*Rosellinia necatrix*-PcPCL1606 interaction. HPR production by PcPCL1606 was shown to play a key role in the persistence and colonization ability on the avocado roots, while non-HPR-producing mutants show lower colonization levels on the avocado roots and on *R. necatrix* CH53 hyphae (Calderón et al., 2014b). Other recent studies suggested additional roles of the antibiotics produced by a *P. chlororaphis* strain. Thus, the role of PHZ production by *P. chlororaphis* in biofilm formation has been extensively studied, confirming its involvement in biofilm formation but reducing its role during biocontrol (Maddula et al., 2008; Selin et al., 2010, 2012).

The objective of this study was to elucidate the role of HPR production in the ability of PcPCL1606 to form biofilms and determine the presence of this antifungal antibiotic affects the development and biofilm structures.

## Materials and Methods

### Bacterial Strains and Culture Conditions

The wild-type strain PcPCL1606 and the different derivative strains used in this study (Table 1) were grown on tryptone-peptone-glycerol (TPG) medium (Calderón et al., 2013). The bacterial strains were stored at -80°C in LB with 10% dimethyl sulfoxide. The media was supplemented with kanamycin (50 μg/mL) and gentamicin (30 μg/mL), when necessary.

**Table 1:** Bacterial strains used in this study.

Strain Relevant characteristics^a^			Reference
***Bacterial strains***			
***Pseudomonas chlororaphis***			
PCL1606		Wild-type, isolated from Spanish avocado rhizosphere, HPR +++	Cazorla et al., 2006
*darA-*		PCL1606 derivative insertional mutant in *darA* gene, HPR -, Km^r^	Calderón et al., 2013
*darB-*		PCL1606 derivative insertional mutant in *darB* gene, HPR -, Km^r^	Calderón et al., 2013
*darC-*		PCL1606 derivative insertional mutant in *darC* gene, HPR +, Km^r^	Calderón et al., 2013
*darS-*		PCL1606 derivative insertional mutant in *darS* gene, HPR ++, Km^r^	Calderón et al., 2013
*darR-*		PCL1606 derivative insertional mutant in *darR* gene, HPR ++, Km^r^	Calderón et al., 2013
GacS-		PCL1606 derivative insertional mutant in *gacS* gene, HPR -, Km^r^	Cazorla et al., 2006
Δ*darB*		PCL1606 derivative deletional mutant in *darB* gene, HPR -	This study
ComB		Δ*darB* transformed with the plasmid pCOMB. HPR +++, Gm^r^ and Km^r^	This study
***Fungal strains***			
***Fusarium oxysporum* f. sp. *radicis-lycopersici***			
ZUM2407		Causes crown and foot rot of tomato	IPO-DLO Wageningen, The Netherlands
***Rosellinia necatrix***			
CH53		Wild-type, isolated from avocado root rot, High virulence	Pérez-Jiménez, 1997
**Plasmids**			
pCOMB		*dar*B gene cloned into pBBR1MCS-5 used for complementing mutation on strain Δ*darB*, Gm^r^	Calderón et al., 2013
pBAH8		pBBR1MCS-5-containing PA1/04/03-gfp mut3-To-T1; Gm^r^	Huber et al., 2002
pGEM^®^-T Easy Vector		Linearized vector with single 3’-terminal thymidine at both ends	Promega


*^a^HPR: Production of 2-hexyl, 5-propyl resorcinol detected by thin-layer chromatography plates (Cazorla et al., 2006). +++ = HPR production level of the wild-type strain *P. chlororaphis* PCL1606; ++ = ½ of HPR production; + = ¼ of HPR production; - = no production (Calderón et al., 2014a). Antibiotic resistance: Km^*r*^ = Kanamycin, Gm^*r*^ = Gentamycin. IPO-DLO: Institute for Plant Protection – Agriculture Research Department.*

A collection of insertional mutants in each of the *dar* genes was available from earlier studies (Calderón et al., 2013; Table 1). However, to use genetically clean mutants of the biosynthetic *dar* genes, a deletional mutant in the *darB* biosynthetic gene (Δ*darB*) was constructed as previously described (Matas et al., 2014). Briefly, upstream and downstream fragments of the *darB* region to be deleted were cloned into the pGEM-T Easy Vector^®^ as described by Matas et al. (2014). Later, the *npt*II kanamycin resistance gene obtained from pGEM-T-KmFRT-*Hind*III was introduced into the plasmid, yielding pGEM-T-Δ*darB*-Km. Finally, each plasmid was transformed by electroporation into PcPCL1606 for marker exchange mutagenesis. Screening and verification of the mutants was conducted as previously described (Matas et al., 2014).

For visualization using confocal laser scanning microscopy (CLSM), the bacterial strains listed in Table 1 were transformed with the plasmid pBAH8 (Huber et al., 2002), which expresses the *gfp* gene (green) as previously described (Calderón et al., 2014b). The phenotypic characteristics of each transformed bacterial strain were analyzed (growth on minimal and rich medium, antagonism, and HPR production) using the procedures described below.

### HPR Production and Antagonism

To check the proper phenotypes, HPR production and fungal antagonism were tested for the wild-type and different derivative strains as previously described (Cazorla et al., 2006). Briefly, for HPR production, cell-free supernatants from 5-day-old liquid KB cultures of the test strain were extracted using chloroform/methanol (2:1, v/v). The organic fractions were dried and resuspended in 100 μL acetonitrile. Fifty microliters of the extractions were fractionated by thin layer chromatography (TLC) using silica RP-18F_254S_ TLC plates (Merck AG, Darmstadt, Germany) in chloroform:acetone (9:1, v/v). After drying, the chromatogram was visualized under UV light at 254 nm, and the *Rf* values were calculated. Antibiotic production was also determined by spraying these TLC plates with diazotized sulfanilic acid and watching for a characteristic color change (Whistler et al., 2000). Spots with a *Rf* value of approximately 0.9–0.95 that were brown to dark green in color were considered positive for HPR. The wild-type strain PcPCL1606 was used as a reference for antibiotic production (Cazorla et al., 2006). The HPR production was quantified as previously described (Calderón et al., 2014a).

The antagonistic activity was tested *in vitro* as previously described (Geels and Schippers, 1983; Cazorla et al., 2006) using the fungal pathogens *R. necatrix* CH53 and *Fusarium oxysporum* f. sp. *radicis-lycopersici* ZUM2407 (Table 1). Experiments were performed on KB and PDA plates as follows: a 0.6-cm-diameter mycelial disk from a 5-day-old fungal culture was placed in the center of a Petri dish at 24°C, and the bacterial strain was inoculated at a distance of approximately 3 cm from the fungus. The antagonistic strain PcPCL1606 was used as a control. The fungal inhibition was recorded after 5 days of growth.

### Swimming Motility Assay

For the swimming analysis, the bacteria were inoculated with a sterile toothpick into the center of a 0.3% agar plate with TPG diluted 1/20 in MilliQ water similarly to a method previously described (Dekkers et al., 1998). The plates were analyzed after 24 h of incubation at 25°C. Measurements of the motility circle radius enabled the calculation of the motility area. Five independent experiments were performed.

### Biofilm Adhesion Assay

To test the early phases of biofilm formation, adhesion to an abiotic surface was performed. Biofilm formation was assayed by the ability of the cells to adhere to the wells of 96-well microtiter dishes comprised of polyvinylchloride plastic (PVC, Tissue culture plate 96-well, round bottom suspension cells, Sarstedt) as previously described with modifications (O’Toole and Kolter, 1998). An exponential culture of the bacterial strains (TPG media, 10 h at 25°C) was adjusted to an O.D._600nm_ of 0.08 (10^7^ cfu/mL) with sterile TPG medium. The different test bacterial strains were distributed (100 μL of bacterial suspension) into each well (at least six wells inoculated per strain). After inoculation, the plates were incubated at 25°C for 3 days without movement. A total of 120 μL of a 1% crystal violet solution was added to each well to stain the cells, and the plates were incubated at room temperature for 30 min and rinsed thoroughly and repeatedly with water. Finally, 120 μL of 50% methanol was added to each well to solubilize the crystal violet at room temperature for 20 min. The amount of crystal violet present in each well was determined by absorbance at 595 nm to quantify the biofilm.

### Interface Pellicle Air–Liquid Formation

Pellicle biofilm formation on the surface of a liquid culture was analyzed using liquid TPG media (Calderón et al., 2013). For pre-cultures, each test strain was exponentially grown in TPG medium during 10 h. The bacterial concentration was adjusted with TPG medium to an O.D._600nm_ of 0.8 (10^8^ cfu/mL) with TPG media, and 10 μL of the adjusted suspension was used to inoculate 1 mL of TPG in 24-well plates to a final O.D._600nm_ of 0.08. The inoculated plates were incubated without agitation at 25°C for 6 days, and the presence of the characteristic pellicle was then reported (Friedman and Kolter, 2004).

For chemical complementation assays, PcPCL1606 cultures were grown in a 200 mL TPG medium for 28 h at 25°C under orbital shaking (150 rpm), and the production and accumulation of HPR were confirmed as previously described (Calderón et al., 2014a). The cultures were centrifuged, sterilized by filtration (0.22-μm filter), and the cell-free supernatant was collected. One hundred milliliters of PcPCL1606 cell-free supernatant was added to 100 mL of the TPG medium as previously described (Mori et al., 2017). The mixture was distributed in 24-well plates. Finally, 10 μL of a cell suspension (0.8 at O.D._600nm_) of Δ*darB*, COMB, or PcPCL1606, was inoculated in the plate wells containing the cell-free supernatant/TPG mixture, and interface pellicle air–liquid formation assays were conducted. This same experiment was repeated using the wild-type PcPCL1606 and Δ*darB* GFP-tagged to visualize the pellicle structure using scanning confocal laser microscopy. After 6 days of growth, the pellicle was separated from the well and carefully removed for microscopic visualization. As a negative control, this experiment was also performed using the supernatant of the Δ*darB* mutant. To avoid the possibility of other compounds that may play a role in regulating biofilm formation, the culture supernatant of the Δ*darB* mutant on biofilm formation in the wild-type strain was tested.

The same experiment of chemical complementation was performed using *gfp*-tagged strains (wild-type and Δ*darB*-derivative strain), in order to visualize the pellicle using CLSM. The obtained pellicle was carefully removed and mounted on a glass slide for observation. When no pellicle was observed, the culture was sampled to confirm the presence of planktonic *gfp*-tagged strains. All the experiments were performed in biological triplicate.

### Colony Morphology Assay

The wild-type strain and the derivative mutants were plated on 1% TPG agar plates to obtain single isolated colonies to observe the colony morphology and polysaccharide production, measured as Congo red binding ability of the colony. One percent TPG agar medium was supplemented with 40 μg/mL Congo red (CR; Sigma) and 20 μg/mL Coomassie brilliant blue (CB; Sigma). Ten microliters of cell suspension with an O.D._600nm_ of 0.8 (10^8^ cfu/mL) was spotted onto the different agar plates and grown at 25°C during 5 days (Ramos et al., 2010). Images were captured using a fluorescence stereomicroscope AZ-100. All the assays were repeated three times with independent bacterial cultures.

To confirm the chemical complementation, cells-free supernatants were obtained as described above. To prepare the complementation plates, 100 mL of cell-free supernatant was added to 100 mL the TPG agar media (with a double agar concentration), supplemented with CR and CB adjusted to a final concentration of 40 and 20 μg/mL, respectively. The wild-type strain PcPCL1606 and the Δ*darB* mutant were assayed. Ten microliters of the test cultures (0.8 at O.D._600nm_) was inoculated in the TPG and in complementation plates. The characteristics of the bacterial colonies were observed after 5 days of growth. All the experiments were performed in triplicate.

### Biofilm Architecture

To determine if the architecture of a biofilm was finally altered at the early stages of maturation, a flow experiment was performed. Biofilms were grown in flow cells supplied with AB minimal media (Clark and Maaløe, 1967). The flow system was assembled and prepared as described previously (Christensen et al., 1999). Briefly, the flow channels were inoculated with different GFP-tagged *P. chlororaphis* cultures grown at a low cell density (OD_600nm_) in AB minimal media supplemented with 1 mM citrate as the carbon source. The medium flow was maintained at a constant rate of 0.2 mm/s using a Watson-Marlow 205S peristaltic pump. The incubation temperature was 25°C. Microscopic inspection and image acquisition were performed using a confocal laser scanning microscope (DM5500Q; Leica) equipped with a 40/1.3 or a 63/1.4 oil objective. The images captured were analyzed with the Leica Application Suite (Mannheim, Germany) and the Imaris software package (Bitplane, Switzerland). Images were prepared for publication using CorelDraw (Corel Corporation) and PowerPoint (Microsoft) software.

### Statistical Methods

The data were statistically analyzed using an analysis of variance (Sokal and Rohlf, 1986), followed by Fisher’s least significant difference test (*P* = 0.05) using the SPSS 24 software (SPSS Inc., Chicago, IL, United States). All the experiments were performed at least three times independently.

## Results

### Phenotypic Characterization of the Mutant Derivatives

The analysis of the derivative strains used in this study was performed using molecular and phenotypical assays, such as antagonism and the production of HPR by the TLC test. Our results showed that the *darA-*, Δ*darB*, *darB-*, and *gacS-* mutants did not produce HPR. HPR production was detected in the *darC-*, *darS-*, and *darR-* mutants but at a lower amount when compared with the wild-type strain. The complemented strain displayed HPR production at levels similar to that observed for the wild-type strain (Table 1). Similar results were obtained when antagonism was analyzed, with an absence of antagonistic activity for *darA-*, Δ*darB*, *darB-*, and *gacS-*, moderate antagonism for *darC-*, *darS-*, and *darR-*, and strong antagonism for the complemented strain (data not shown).

### Swimming Motility Assay

**FIGURE 1:**
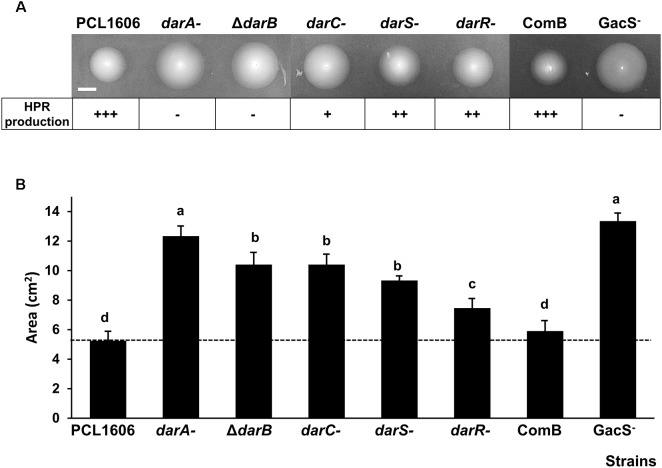
Swimming motility of *P. chlororaphis* PCL1606 and its derivatives after 24 h of incubation at 25°C. **(A)** Swimming haloes of *P. chlororaphis* PCL1606 and its derivative strains. HPR production is indicated. +++ = HPR production level of the wild-type strain *P. chlororaphis* PCL1606; ++ = ½ of HPR production compared to the wild-type; + = ¼ of HPR production compared to the wild-type; – = no production. Scale bar indicates 1 cm. **(B)** Average swimming halo area (cm^2^) and standard deviations of five independent experiments are presented. Different letters indicate statistically significant differences (*P* > 0.05).

The swimming motility was quantified by measuring the swimming area in the TPG medium agar plate (Figure 1A). All the non-HPR-producing mutants used in this study (Table 1) exhibited increased motility when compared to the wild-type strain. The mutants showed a swimming area of 7.46–13.35 cm^2^, while the wild-type strain covered an area of 5.25 cm^2^ (Figure 1B).

The introduction of a plasmid containing the *darB* gene into the Δ*darB* mutant (COMB strain) restored the HPR production and decreased motility (5.90 cm^2^), reaching motility levels similar to those displayed by the wild-type strain when compared to those motility values of the Δ*darB* mutant (12.2 cm^2^) affected in HPR production.

The mutant in GacS displayed the highest motility of all the strains assayed.

### Adhesion Assays to PVC Microtiter Wells

**FIGURE 2:**
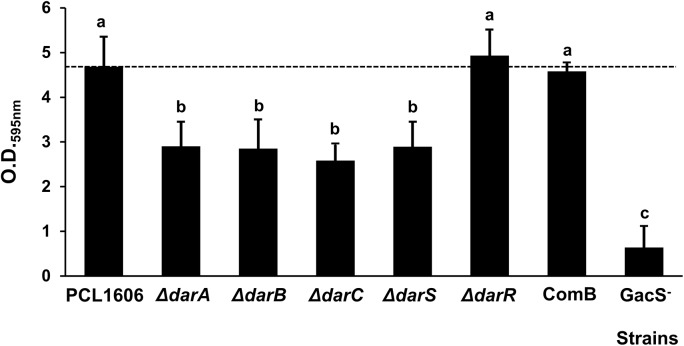
Biofilm adhesion assay of *P. chlororaphis* PCL1606 and its derivatives strains. The cultures were grown in 96-well microtiter dishes comprised of PVC containing TPG media for 3 days at 25°C. Biofilm formation, indicated by crystal violet staining, was measured at an absorbance of 595 nm. Different letters indicate statistically significant differences (*P* > 0.05).

Mutants with defects in the biosynthetic genes (*darABC*), and the transcriptional regulators *darS* and *GacS* were found to be impaired in their adhesion to the PVC surfaces (Figure 2). Impairment in the HPR production strains, such as the *darC-* or *darS-* mutants, produces lower amounts of HPR than the wild-type strain, and displayed significantly lower adhesion levels. Only the *darR-* mutant (producing half of the normal amount of HPR) and the COMB strain displayed the same adhesion levels as the wild-type strain.

In those experiments, the derivative mutant in GacS showed almost no adhesion to PVC with the lower values of absorbance at O.D._595nm_.

### Interface Liquid–Air Pellicle Formation

**FIGURE 3:**
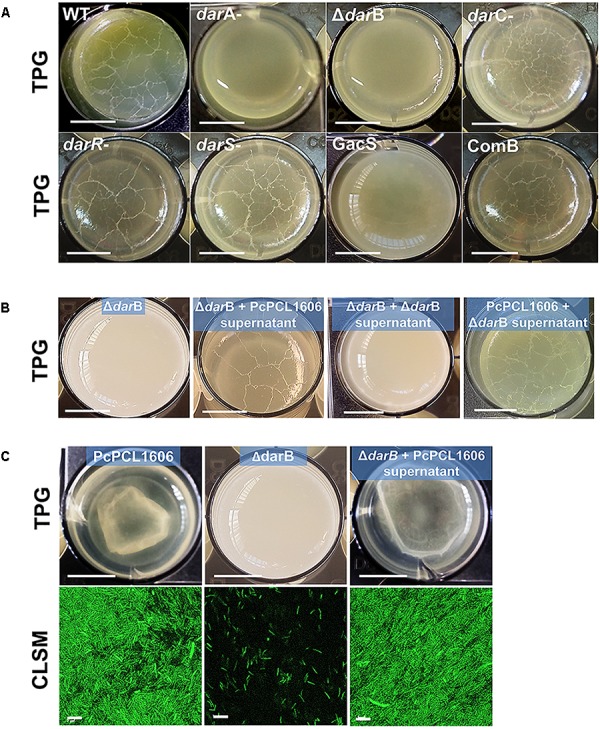
Interphase air–liquid pellicle formation. **(A)** Pellicle formation of *P. chlororaphis* PCL1606 and its derivative mutants. Strains were inoculated (final O.D._600nm_ of 0.08) and grown on TPG media in 24-well plates without agitation at 25°C for 6 days. **(B)** A similar experiment but using cell-free supernatants of PcPCL1606 mixed with TPG as growth media in order to demonstrate the pellicle recovery in the liquid medium by the defective mutant Δ*darB*. **(C)** The same experiment as **B**, using GFP-tagged strains to visualize the pellicle using confocal scanning laser microscopy. Size of the bars in the microwell pictures: 5 mm; bars of confocal laser scanning microscopy: 5 μm.

All the HPR producing strains (wild-type PcPCL1606 strain and derivatives *darC-*, *darS-*, *darR-*, and COMB) formed pellicles in the liquid–air interface, while no pellicle formation was observed with the non-HPR producing strains, such as *gacS-* and the *darA-* and Δ*darB* mutants impaired in HPR production (Figure 3A).

When the chemical complementation was assayed, the derivative strain *ΔdarB* (non-pellicle former strain on TPG) was able to form pellicle when growing on TPG media amended with sterile spent TPG media of a PcPCL1606 culture that had been growing previously (Figure 3B), but no pellicle was observed when grown on TPG amended with sterile supernatants from a Δ*darB* culture. The wild-type strain forms the typical pellicle when growing on the culture supernatant of the Δ*darB* mutant, excluding the presence of other compounds that could play a role in biofilm formation.

Because chemical complementation experiments demonstrated the presence of pellicle when HPR production has taken place in the media, a similar assay was performed using gfp-tagged strains. The pellicle formed by the wild-type strain PcPCL1606-GFP (HPR producing strain) and Δ*darB*-GFP (non-HPR producing strain) showed no differences when compared under CLSM with fluorescent cells tightly grouped when PcPCL1606-GFP is grown in TPG and when Δ*darB*-GFP is grown in the liquid media amended with the cell-free supernatants from PcPCL1606 (Figure 3C). The mutant Δ*darB*-GFP, which is defective for HPR production, showed no biofilm and only dispersed viable planktonic cells were observed.

### Colony Morphology on Agar-Congo Red

**FIGURE 4:**
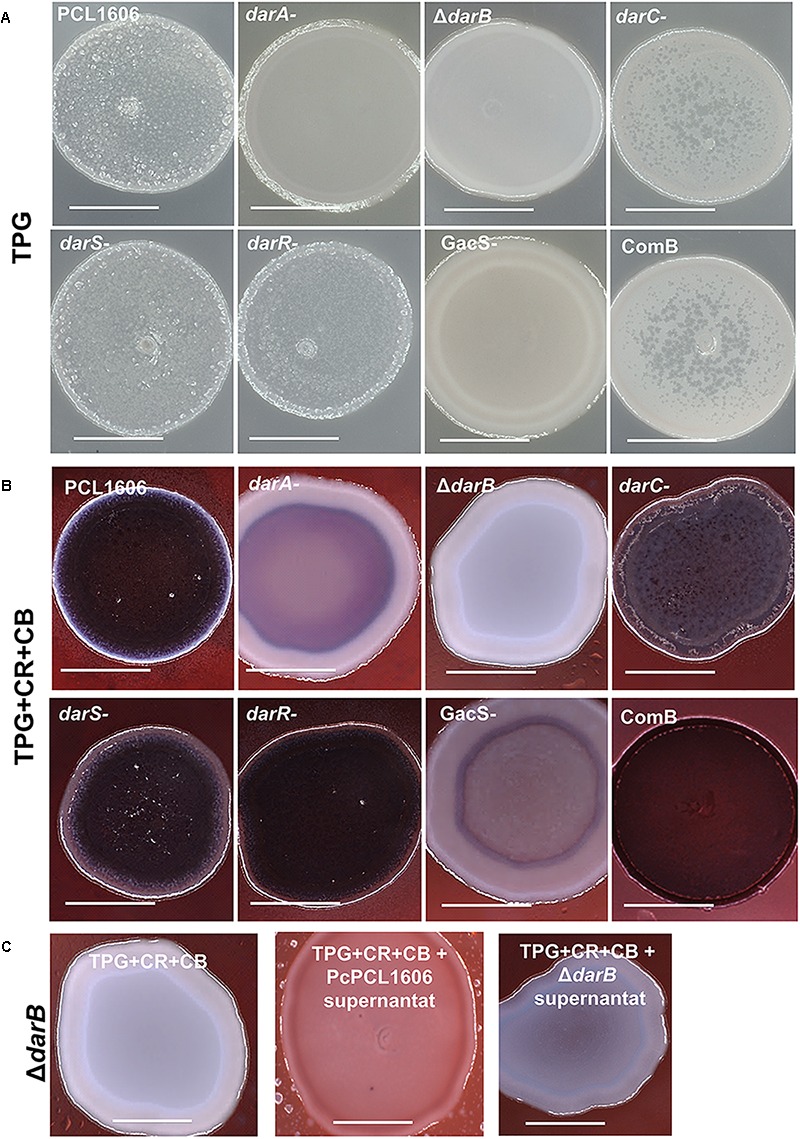
Colony morphology assay and analysis of Congo red binding. *P. chlororaphis* PCL1606 and its derivative mutants when were grown on **(A)** 1% TPG agar plates and **(B)** 1% TPG agar plates containing 40 μg/mL Congo red and 20 μg/mL Coomasssie brilliant blue; **(C)** a similar experiment but using TPG plate media amended with cell-fee supernatants of PcPCL1606 (1:1) to demonstrate the restoration in colony morphology of the defective mutant Δ*darB*. The plates were incubated at 25°C for 5 days. Size of the bar is 5 mm.

In the colony morphology assay, cell suspensions are spotted on 1% TPG agar-solidified media supplemented with CR and CB (Friedman and Kolter, 2004; Madsen et al., 2015). In this media, the wild-type strain and the HPR producing strains are characterized by a wrinkled surface and a strong red color due to the Congo red binding, indicating the polysaccharide production. However, the derivative mutants affected in HPR production (*darA-* and *ΔdarB*) and in the transcriptional regulator *GacS* (*gacS-*) showed a smooth surface (on TPG media) and a lack of red colors on TPG media with CR and CB (Figure 4A,B).

In a similar assay, but using a solid agar plate supplemented with cell-free PcPCL1606 supernatant (50% v/v), the *ΔdarB* derivative mutant was able to accumulate some red color in the colony, partially complementing the mutant phenotype and exhibiting more similar to the wild-type colony (Figure 4C).

### Biofilm Architecture

**FIGURE 5:**
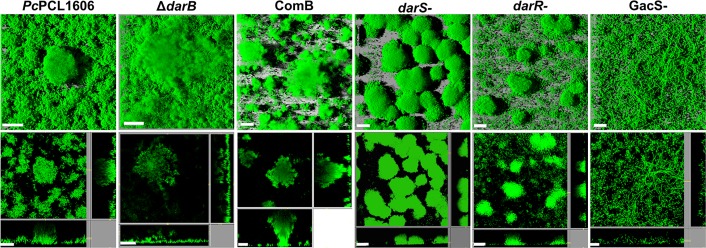
Biofilm architecture of the strains tested. Flow cells were inoculated with a low-density culture of *P. chlororaphis* PCL1606, the non-HPR producing derivative strain in *darB* gene (Δ*darB*), the complemented Δ*darB* derivative strain (ComB), and the transcriptional regulators (Δ*darS*, Δ*darR*, and *GacS*) derivative mutants, using AB minimal media supplemented with 1 mM citrate. All the strains were transformed with GFP plasmid for visualization. Biofilm formation was assayed using by confocal laser scanning microscopy. The large frames show the top view, whereas the right and lower frames show vertical sections through the biofilm. Scale bars: 20 μm.

Biofilm architecture was analyzed at the initial stage of biofilm maturation using flow-through flow cells chambers (Figure 5). We found differences between the wild-type and the different derivatives strains tested in this experiment, in which the primary difference is related to HPR production. As shown in Figure 5, we observed that the wild-type strain, which produces a high amount of HPR (Calderón et al., 2014a), was dominated by large microcolonies forming the characteristics of mushroom-shaped structures and characterized by a completely covered surface, while the transcriptional regulator (*darSR*), which produced half of the HPR in comparison to the wild-type strain (Calderón et al., 2014a), only a few cells colonized the void space between the microcolonies. The primary difference with respect to the wild-type strain was observed with the non-HPR-producing mutants Δ*darB* and *gacS-*. After 3 days of growth, the Δ*darB* strain had formed flat and unstructured colonies with a low volume/area biofilm ratio, and with the *gacS-* mutant no colonies, but filamentous cells were observed.

## Discussion

Antibiotics are widely studied in plant beneficial *Pseudomonas* species, since they are considered to be essential for the biocontrol ability against different soilborne phytopathogenic fungi (Haas and Keel, 2003; Raaijmakers and Mazzola, 2012). However, there are also additional roles for the bacterial antibiotics, which can provide additional ways of participation in the bacterial interaction with the pathogen, such as serving as signals molecules regulating crucial phenotypes (Morohoshi et al., 2013; Brameyer et al., 2015).

Plant-associated pseudomonads have often been studied as model microorganisms for the microbial interaction with plant roots (Lugtenberg and Dekkers, 1999; Lugtenberg et al., 2001). In addition, some colonizing rhizobacteria have been found to form dense biofilm-like structures that occupy the rhizoplane, especially at the junctions between the epidermal root cells (Chin-A-Woeng et al., 1997; Normander et al., 1999; Cassidy et al., 2000; Ramos et al., 2000; Villacieros et al., 2003). However, biofilm formation is considered to be a complex process, and different strains could develop different biofilm architectures, depending also depending on the environmental conditions (Petrova and Sauer, 2012).

PcPCL1606 synthesizes HPR in addition to other antifungal compounds. HPR is crucial in the biocontrol ability of this strain against different soilborne phytopathogenic fungi (Cazorla et al., 2006; Calderón et al., 2013). However, the antifungal activity is not the only role for HPR in PcPCL1606 biology. Previous results showed that non-HPR producing mutants of PcPCL1606 displayed lower root colonization levels (Calderón et al., 2014b), suggesting a possible role of HPR in biofilm formation, similar to what has been reported for the antifungal compounds PHZs. PHZs can participate in the interaction with the pathogen, participating in cell-to-cell communication as signal molecules with key roles in biofilm formation (Zhang and Pierson, 2001; Dietrich et al., 2006; Morohoshi et al., 2013). This phenotype has been explored in other studies, and it has been proposed that antibiotics can play multiple, concentration dependent roles. At lower concentrations, antibiotics could act as signaling molecules capable of modulating gene expression, while at higher concentrations, they function as inhibitors (Davies et al., 2006; Fajardo and Martínez, 2008).

Swimming motility is involved in biofilm formation (Watnick and Kolter, 2000). The swimming ability of PcPCL1606 could play an essential role during the first stage of biofilm formation through cell/cell and cell/surface interaction, as has been previously described in other *Pseudomonas* spp. (Li et al., 2015). Thus, swimming motility in the *dar* and *gacS-* derivative mutants of PcPCL1606 were analyzed. The *gacS-* mutant showed the expected phenotype of hypermotility, also observed for the *gacS-* derivatives mutant of *Pseudomonas fluorescens* F113, resulting in an increasing swimming motility (Martínez-Granero et al., 2006). Curiously, HPR-defective mutants also showed an increase in swimming motility, consistent with the hypothesis that HPR is involved in biofilm formation, since mobile and sessile are opposite biological states (Petrova and Sauer, 2012).

Thus, the opposite phenotype should be expected for these strains when the adhesion assay is performed. Adhesion is considered to be one of the first steps in biofilm development (O’Toole and Kolter, 1998). When the *dar* and *gacS* mutants were tested, non-HPR producers showed a reduced adhesion to the PVC surfaces with the exception of the *darR* mutant (Figure 2). Since this mutant is impaired in a regulatory gene, additional biological processes could be affected that may explain its similar phenotype to the wild-type strain (Calderón et al., 2014a). Similar results have also been observed for the production of antifungal PHZ production in other strains of *P. chlororaphis*, such as when the PHZ-1-carboxylic acid (PCA) and 2-hydroxy-PCA (2-OH-PCA) ratio is altered, the initial biofilm attachment is affected (Maddula et al., 2008; Selin et al., 2010).

When colony morphology analyses were performed, defective strains in HPR production and a defective mutant in GacS showed an extracellular component deficit. The lack of red dye in the bacterial colony when growing in the presence of CR revealed the lack of exopolysaccharides that usually dye red with CR (Friedman and Kolter, 2004). This lack of polysaccharides usually leads to inconsistency of the extracellular matrix (Madsen et al., 2015). This fact is reflected by the absence of pellicle formation in the air–liquid interphase. This pellicle is produced by the cultures of all HPR-producing strains. The non-pellicle former Δ*darB* mutant restores biofilm formation when it is genetically and chemically complemented at a level that is undistinguishable from the wild-type strain.

Finally, to unravel if the lack of HPR production can affect the development of a more mature biofilm, flow chamber experiments were conducted. When the biofilm architecture was observed on the wild-type strain and COMB derivative strain, the formation of a large and distinctive mushroom-like cluster with a high biofilm density around the colonies was observed. However, mutants unable to produce HPR, such as Δ*darB* or GacS-, showed the presence of a slightly disorganized layer of bacteria, probably due to a modification of the biofilm matrix, indicated by the absence of exopolysaccharide production on the CR plates, similar to the results previously observed for *Pseudomonas aeruginosa* (Colvin et al., 2011). The intermediate stages of HPR production, such as the *darS-* and *darR-* mutants, give rise to intermediate structures of biofilm, observing a direct relationship between HPR quantity and biofilm consistency. Similar results were described previously in *P. chlororaphis* strain 30-84, in which the biofilm formation is dependent on the type of PHZs produced. The production of only PCA or 2-OH-PCA resulted in a substantially thicker mushroom-shaped cluster with a higher cell density than the wild-type strain (Maddula et al., 2008).

Therefore, it is straightforward to conclude that HPR is a molecule that has a role in biofilm organization in PcPCL1606, and its lack resembles to the phenotype resulting from the inactivation of the regulatory system GacS-GacA (Choi et al., 2007). These facts reinforce the previously reported relation of the biosynthesis of HPR and its regulation by the *darSR* genes also regulated at a higher level by the two-component regulatory system *gacS*-*gacA* (Calderón et al., 2014a). The results presented in this study indicate the direct involvement of HPR in biofilm development by PcPCL1606 for the first time. In addition, the requirement for HPR has also been shown to be directly involved in the production of polysaccharide compounds in the biofilm matrix. Some *P. chlororaphis* strains have shown evidences that they of harbor an additional quorum-sensing system sharing genes with the PHZ regulation genes *phzI* and *phzR* (Zhang and Pierson, 2001), also required for biofilm formation (Maddula et al., 2008). However, PcPCL1606 is an atypical *P. chlororaphis*, and it does not produce any type of PHZs. HPR is the main antifungal molecule produced by PcPCL1606.

Finally, the fact that the dialkylresorcinols and cyclohexanediones could be a cell-to-cell communication signal molecule instead of AHL (Brameyer et al., 2015) strengthen the hypothesis that HPR acts as a regulatory signal for phenotypes, such as biofilm formation via exopolysaccharide production.

## Author Contributions

CC, LE, and FC designed the experiments. CC, ST, EA, ZH-P, and GC-O performed the experiments. CC, LE, and FC analyzed the results and wrote the manuscript. All the authors read and approved the final manuscript.

## Conflict of Interest Statement

The authors declare that the research was conducted in the absence of any commercial or financial relationships that could be construed as a potential conflict of interest.

## References

[B1] Brameyer S. Kresovic D. Bode H. B. Heermann R. (2015). Dialkylresorcinols as bacterial signalling molecules. *Proc. Natl. Acad. Sci.* 112 572–577. 10.1073/pnas.1417685112 25550519 PMC4299209

[B2] Calderón C. E. Carrión V. J. de Vicente A. Cazorla F. M. (2014a). darR and darS are regulatory genes that modulate 2-hexyl, 5-propyl resorcinol transcription in *Pseudomonas chlororaphis* PCL1606. *Microbiology* 160 2670–2680. 10.1099/mic.0.082677-0 25234473

[B3] Calderón C. E. de Vicente A. Cazorla F. M. (2014b). Role of 2-hexyl, 5-propyl resorcinol production by *Pseudomonas chlororaphis* PCL1606 in the multitrophic interactions in the avocado rhizosphere during the biocontrol process. *FEMS Microbiol. Ecol.* 89 20–31. 10.1111/1574-6941.12319 24641321

[B4] Calderón C. E. Pérez-garcía A. de Vicente A. Cazorla F. M. (2013). The dar genes of *Pseudomonas chlororaphis* PCL1606 are crucial for biocontrol activity via production of the antifungal compound 2-hexyl, 5-propyl resorcinol. *Mol. Plant-Microbe Interact.* 26 554–565. 10.1094/MPMI-01-13-0012-R 23547906

[B5] Calderón C. E. Ramos C. de Vicente A. Cazorla F. M. (2015). Comparative genomic analysis of *Pseudomonas chlororaphis* PCL1606: insight into antifungal traits involved in biocontrol. *Mol. Plant-Microbe Interact.* 28 249–260. 10.1094/MPMI-10-14-0326-FI 25679537

[B6] Cassidy M. B. Leung K. T. Lee H. Treyors J. T. (2000). A comparison of enumeration methods for culturable *Pseudomonas fluorescens* cells marked with green fluorescent protein. *Microbiol. Methods* 40 135–145. 10.1016/S0167-7012(99)00131-1 10699669

[B7] Cazorla F. M. Duckett S. Bergström E. Noreen S. Odijk R. Lugtenberg B. J. J. (2006). Biocontrol of avocado *Dematophora* root rot by antagonistic *Pseudomonas fluorescens* PCL1606 correlates with the production of 2-hexyl, 5-propyl resorcinol. *Mol. Plant-Microbe Interact.* 19 418–428. 10.1094/MPMI-19-0418 16610745

[B8] Chin-A-Woeng T. F. C. De Priester W. Van Der Bij A. J. Lugtenberg B. J. J. (1997). Description of the colonization of a gnotobiotic tomato rhizosphere by *Pseudomonas fluorescens* biocontrol strain WCS365, using scanning electron microscopy. *Mol. Plant Microbe Interact.* 10 79–86. 10.1094/MPMI.1997.10.1.79

[B9] Choi K. S. Veeraraqouda Y. Cho K. M. Lee S. O. Jo G. R. Lee K. (2007). Effect of gacs and gaca mutation in colony architecture, surface motility, biofilm formation and chemical toxicity in *Pseudomonas* sp. KL28. *J. Microbiol.* 45 492–498. 18176530

[B10] Christensen B. B. Sternberg C. Andersen J. B. Palmer R. J. Jr. Nielsen A. T. Givskov M. (1999). Molecular tools for study of biofilm physiology. *Methods Enzymol.* 310 20–42. 10.1016/S0076-6879(99)10004-1 10547780

[B11] Clark D. J. Maaløe O. (1967). DNA replicaton and the division cycle in *Escherichia coli*. *J. Mol. Biol.* 23 99–112. 10.1016/S0022-2836(67)80070-6

[B12] Colvin K. M. Gordin V. D. Murakami K. Borlee B. R. Wozniak D. J. Wong G. C. L. (2011). The pel polysaccharide can serve a structural and protective role in the biofilm matrix of *Pseudomonas aeruginosa*. *PLoS Pathog.* 7:e1001246. 10.1371/journal.ppat.1001264 21298031 PMC3029257

[B13] Davies J. Spiegelman G. B. Yim G. (2006). The world of subinhibitory antibiotic concentrations. *Curr. Opin. Microbiol.* 9 445–453. 10.1016/j.mib.2006.08.006 16942902

[B14] Dekkers L. C. de Weger L. A. Wijffelman C. A. Spaink H. P. Lugtenberg B. J. J. (1998). A two component system plays an important role in the root colonising ability of *Pseudomonas fluorescens* strain WCS365. *Mol. Plant-Microbe Interact.* 11 45–56. 10.1094/MPMI.1998.11.1.45 9425686

[B15] Dietrich L. E. Price-Whelan A. Petersen A. Whiteley M. Newman D. K. (2006). The phenazine pyocyacin is a terminal signaling factor in the quorum sensing network of *Pseudomonas aeruginosa*. *Mol. Microbiol.* 61 1308–1321. 10.1111/j.1365-2958.2006.05306.x 16879411

[B16] Faheem M. Raza W. Jun Z. Shabbir S. Sultana N. (2015). Characterization of the newly isolated antimicrobial strain *Streptomyces goshikiensis* YCXU. *Sci. Lett.* 3 94–97.

[B17] Fajardo A. Martínez J. L. (2008). Antibiotics as signals that trigger specific bacterial responses. *Curr. Opin. Microbiol.* 11 161–167. 10.1016/j.mib.2008.02.006 18373943

[B18] Friedman L. Kolter R. (2004). Genes involved in matrix formation in *Pseudomonas aeruginosa* PA14 biofilms. *Mol. Microbiol.* 51 675–690. 10.1046/j.1365-2958.2003.03877.x 14731271

[B19] Geels F. P. Schippers G. (1983). Selection of antagonistic fluorescent *Pseudomonas* spp., and their root colonization and persistence following treatment of seed potatoes. *Phytopathology* 108 193–206. 10.1111/j.1439-0434.1983.tb00579.x

[B20] Gross H. Loper J. E. (2009). Genomics of secondary metabolism in *Pseudomonas* spp. *Nat. Prod. Rep.* 26 1408–1446. 10.1039/B817075B 19844639

[B21] Haas D. Keel C. (2003). Regulation of antibiotic production in root-colonizing *Pseudomonas* spp. and relevance for biocontrol of plant disease. *Annu. Rev. Phytopathol.* 41 117–153. 10.1146/annurev.phyto.41.052002.095656 12730389

[B22] Huber B. Riedel K. Köthe M. Givskov M. Molin S. Eberl L. (2002). Genetic analysis of functions involved in the late stages of biofilm development in *Burkholderia cepacia* H111. *Mol. Microbiol.* 2 411–426. 10.1046/j.1365-2958.2002.03182.x 12406218

[B23] Li Y. Bai F. Xia H. Zhuang L. Xu H. Jin Y. (2015). A novel regulator PA5022 (aefA) is involved in swimming motility, biofilm formation and elastase activity of *Pseudomonas aeruginosa*. *Microbial. Res.* 176 14–20. 10.1016/j.micres.2015.04.001 26070688

[B24] Lugtenberg B. J. J. Dekkers L. Bloemberg G. (2001). Molecular determinants of rhizosphere colonization by *Pseudomonas*. *Anu. Rev. Phytopathol.* 39 461–490. 10.1146/annurev.phyto.39.1.461 11701873

[B25] Lugtenberg B. J. J. Dekkers L. C. (1999). What makes *Pseudomonas* bacteria rhizosphere competent? *Environ. Microbiol.* 1 9–13. 10.1046/j.1462-2920.1999.00005.x 11207713

[B26] Maddula V. S. R. K. Pierson E. A. Pierson L. S. I. I. I. (2008). Altering the ratio of phenazines in *Pseudomonas chlororaphis* (*aureofaciens*) strain 30-84: effects on biofilm formation and pathogen inhibition. *J. Bacteriol.* 190 2759–2766. 10.1128/JB.01587-07 18263718 PMC2293254

[B27] Madigan T. M. Martinko J. M. (2015). *Brock Biology of Microorganisms*, 14th ed London: Prentice Hall.

[B28] Madsen J. S. Lin Y. C. Squyres G. R. Price-Whelan A. Torio A. S. Song A. (2015). Facultative control of matrix production optimizes competitive fitness in *Pseudomonas aeruginosa* PA14 biofilm models. *Appl. Environ. Microbiol.* 81 8414–8426. 10.1128/AEM.02628-15 26431965 PMC4644639

[B29] Martínez-Granero F. Rivilla R. Martín M. (2006). Rhizosphere selection of highly motile phenotype variants of *Pseudomonas fluorescens* with enhanced competitive colonization ability. *Appl. Environ. Microbiol.* 72 3429–3434. 10.1128/AEM.72.5.3429-3434.2006 16672487 PMC1472367

[B30] Matas I. M. Castañeda-Ojeda M. P. Aragón I. M. Antúnez-Lamas M. Murillo J. Rodríguez-Palenzuela P. (2014). Translocation and functional analysis of *Pseudomonas savastanoi* pv. *savastanoi* NCPPB 3335 Type III secretion system effectors reveals two novel effector families of the *Pseudomonas syringae* complex. *Mol. Plant Microbe Interact.* 27 424–436. 10.1094/MPMI-07-13-0206-R 24329173

[B31] Mori J. F. Ueberschaar N. Lu S. Cooper R. E. Pohnert G. Küsell K. (2017). Sticking together: inter-species aggregation of bacteria isolated from iron snow is controlled by chemical signaling. *ISME J.* 11 1075–1086. 10.1038/ismej.2016.186 28140394 PMC5437920

[B32] Morohoshi T. Wang W. Z. Suto T. Saito Y. Someya N. Ikeda T. (2013). Phenazine antibiotic production and antifungal activity are regulated by multiple quorum-sensing systems in *Pseudomonas chlororaphis* subsp. aurantiaca StFRB508. *J. Biosci. Bioeng.* 116 580–584. 10.1016/j.jbiosc.2013.04.022 23727350

[B33] Morrissey J. P. Cullinane M. Abbas A. Mark G. L. O’Gara F. (2004). “Biosynthesis of antifungal metabolites by biocontrol strains of *Pseudomonas*,” in *Pseudomonas Biosynthesis of Macromolecules and Molecular Metabolism*, ed. Ramos J.-L. (New York, NY: Plenum Publishers), 637–670.

[B34] Normander B. Hendriksen N. B. Nybroe O. (1999). Green fluorescent protein-marked *Pseudomonas fluorescens*: localization, viability, and activity in the natural barley rhizosphere. *Appl. Environ. Microbiol.* 65 4646–4651. 10508101 10.1128/aem.65.10.4646-4651.1999 PMC91619

[B35] Nowak-Thompson B. Philip E. Hammer D. Hill D. S. Staffords J. Torkewitz N. (2003). 2,5-Diakylresorcinol biosynthesis in *Pseudomonas aurantiaca*: novel head-to-head condensation of two fatty acid-derived precursors. *J. Bacteriol.* 185 860–869. 10.1128/JB.185.3.860-869.2003 12533461 PMC142816

[B36] O’Toole G. A. Kolter R. (1998). Initiation of biofilm formation in *Pseudomonas fluorescens* WCS365 proceeds via multiple, pathways convergent signaling: a genetic analysis. *Mol. Microbiol.* 28 449–461. 10.1046/j.1365-2958.1998.00797.x 9632250

[B37] Pérez-Jiménez R. M. (1997). Significant avocado diseases caused by fungi and oomycetes. *Eur. J. Plant Sci. Biotech.* 2 1–24.

[B38] Petrova O. E. Sauer K. (2012). Sticky situations: key components that control bacteria surface attachment. *J. Bacteriol.* 194 2413–2425. 10.1128/JB.00003-12 22389478 PMC3347170

[B39] Raaijmakers J. M. Mazzola M. (2012). Diversity and natural functions of antibiotics produced by beneficial and plant pathogenic bacteria. *Annu. Rev. Phytopathol.* 50 403–424. 10.1146/annurev-phyto-081211-172908 22681451

[B40] Raio A. Reveglia P. Puopolo G. Cimmino A. Danti R. Evidente A. (2017). Involvement of phenazine-1-carboxylic acid in the interaction between *Pseudomonas chlororaphis* subsp. aureofaciens strain M71 and *Seiridium cardinale* in vivo. *Microbiol. Res.* 199 49–56. 10.1016/j.micres.2017.03.003 28454709

[B41] Ramette A. Frapolli M. Saux M. F. L. Gruffaz C. Meyer J. M. Défago G. (2011). *Pseudomonas protegens* sp. nov., widespread plant-protecting bacteria producing the biocontrol compounds 2,4-diacetylphloroglucinol and pyoluteorin. *Syst. Appl. Microbiol.* 34 180–188. 10.2134/jeq2010.0402 21392918

[B42] Ramos C. Molina L. Mølbak L. Ramos J. L. Molin S. (2000). A bioluminiscent derivative of *Pseudomonas putida* KT2440 for deliberate release into the environment. *FEMS Microbiol. Ecol.* 34 91–102. 10.1111/j.1574-6941.2000.tb00758.x 11102686

[B43] Ramos I. Dietrich L. E. P. Price-Whelan A. Newman D. K. (2010). Phenazines affect biofilm formation by *Pseudomonas aeruginosa* in similar ways at various scales. *Res. Microbiol.* 161 187–191. 10.1016/j.resmic.2010.01.003 20123017 PMC2886020

[B44] Raza W. Faheem M. Yousaf S. Rajer F. U. Yamin M. (2013). Volatile and nonvolatile antifungal compounds produced by *Trichoderma harzianum* SQR-T037 suppresssed the growth of *Fusarium oxysporum* f. sp. *niveum*. *Sci. Lett.* 1 21–24.

[B45] Selin C. Fernando W. G. D. de Kievit T. R. (2012). The PhzI/PhzR quorum-sensing system is required for pyrrolnitrin and phenazine production, and exhibits cross-regulation with RpoS in *Pseudomonas chlororaphis* PA23. *Microbiology* 158 896–907. 10.1099/mic.0.054254-0 22262095

[B46] Selin C. Habibian R. Poritsanos N. Athukorola S. N. P. Fernando D. de Kievit T. R. (2010). Phenazines are not essential for *Pseudomonas chlororaphis* PA23 biocontrol of *Sclerotinia sclerotiorum*, but do play role in biofilm formation. *FEMS Microbiol. Ecol.* 71 73–83. 10.1111/j.1574-6941.2009.00792.x 19889032

[B47] Sokal R. R. Rohlf F. J. (1986). *Introduccion a La Bioestadistica.* Barcelona: Dover Publications.

[B48] Villacieros M. Power B. Sánchez-Contreras M. Lloret J. Oruezábal R. I. Martín M. (2003). Colonization behaviour of *Pseudomonas fluorescens* and *Sinorhizobium meliloti* in the alfalfa (*Medicago sativa*) rhizosphere. *Plant Soil* 251 47–54. 10.1023/A:1022943708794

[B49] Wang X. Mavrodi D. V. Ke L. Mavrodi O. V. Yang M. Thomashow L. S. (2015). Biocontrol and plant growth-promoting activity of rhizobacteria from chinese fields with contaminated soils. *Microb. Biotechnol.* 8 404–418. 10.1111/1751-7915.12158 25219642 PMC4408174

[B50] Watnick P. Kolter R. (2000). Biofilm, city of microbes. *J. Bacteriol.* 182 2675–2679. 10.1128/JB.182.10.2675-2679.2000 10781532 PMC101960

[B51] Weller D. M. (2007). *Pseudomonas* biocontrol agents of soilborne pathogens: Looking back over 30 years. *Phytopathology* 97 250–256. 10.1094/phyto-97-2-0250 18944383

[B52] Whistler C. A. Stockwell V. O. Loper J. E. (2000). Lon protease influences antibiotic production and UV tolerance of *Pseudomonas fluorescens* Pf-5. *Appl. Environ. Microbiol.* 66 2718–2725. 10.1128/AEM.66.7.2718-2725.2000 10877760 PMC92065

[B53] Yunus F. Iqbal M. Jabeen K. Kanwal Z. Rashid F. (2016). Antagonistic activity of *Pseudomonas fluorescens* against fungal plant pathogen *Aspergillus niger*. *Sci. Lett.* 4 66–70.

[B54] Zhang Z. Pierson L. S. III (2001). A second quorum-sensing system regulates cell surface properties but not phenazine antibiotic production in *Pseudomonas* aureofaciens. *App. Environ. Microbiol.* 67 4305–4315. 10.1128/AEM.67.9.4305-4315.2001 11526037 PMC93161

